# Supplementary material for: The Genome of Varunaivibrio sulfuroxidans Strain TC8T, a Metabolically Versatile Alphaproteobacterium from the Tor Caldara Gas Vents in the Tyrrhenian Sea
Source: Microorganisms. 2023 May 23;11(6):1366. doi: 10.3390/microorganisms11061366 (PMC10305379; doi:10.3390/microorganisms11061366)
Supplement: Supplementary file 1 [file microorganisms-11-01366-s001.zip › Supplemental_Analyses.pdf]

Supplemental Analyses

Methods

The genome assembly of *Magnetovibrio blakemorei* strain MV-1^T was annotated using the Joint Genome Institute IMG system [1] and the RAST (Rapid Annotation using Subsystem Technology) server [2]. The genome sequence comparison and Blast Dot Plot of *Varunaivibrio sulfuroxidans* strain TC8^T vs. *Magnetovibrio blakemorei* strain MV-1^T were obtained by aligning the two genomes, and blastp comparison of the putative genes identified in both genome within RAST server using default value.

Results

The lower mean value of bastp similarty (57.42 %) (Fig. S1) of the proteins of *V. sulfuroxidans* TC8^T and *M. blakemorei* strain MV-1^T indicates that the two genomes are phylogenetically distant and could explain the high degree of dispersion of the Blast Dot Plot (Fig. S2), along with the high level of genome fragmentation of the *M. blakemorei* genome.

References

1. Markowitz, V.M.; Chen, I.-M.A.; Palaniappan, K.; Chu, K.; Szeto, E.; Grechkin, Y.; Ratner, A.; Jacob, B.; Huang, J.; Williams, P.; et al. IMG: The Integrated Microbial Genomes Database and Comparative Analysis System. *Nucleic Acids Res* **2012**, *40*, D115–D122, doi:10.1093/nar/gkr1044.
2. Aziz, R.K.; Bartels, D.; Best, A.A.; DeJongh, M.; Disz, T.; Edwards, R.A.; Formsma, K.; Gerdes, S.; Glass, E.M.; Kubal, M.; et al. The RAST Server: Rapid Annotations Using Subsystems Technology. *BMC Genomics* **2008**, *9*, 75, doi:10.1186/1471-2164-9-75.

Supplemental Figure Legends

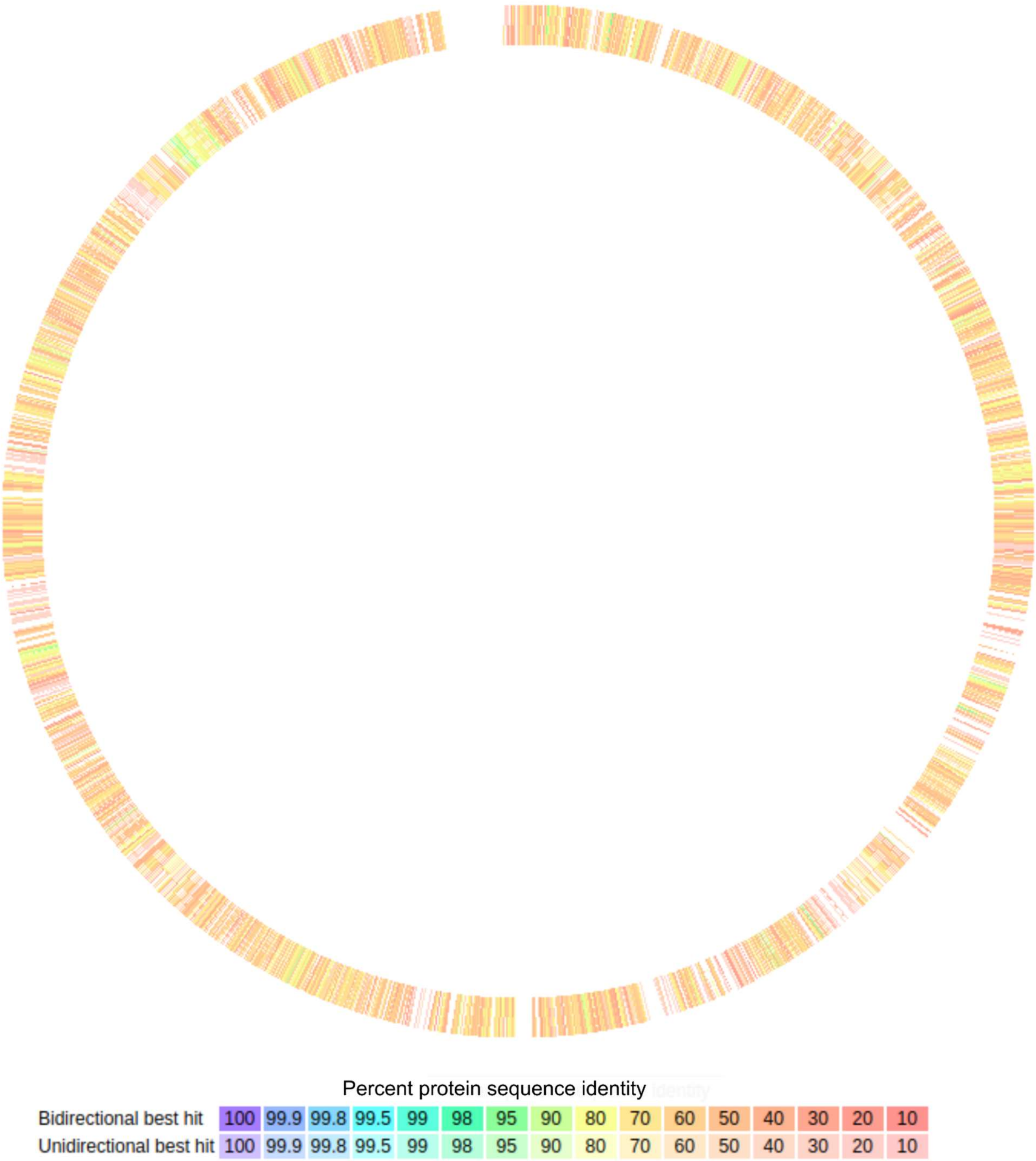

Figure S1. Comparative percent protein sequence identity of *Varunaivibrio sulfuroxidans* strain TC8^T vs. *Magnetovibrio blakemorei* strain MV-1^T.

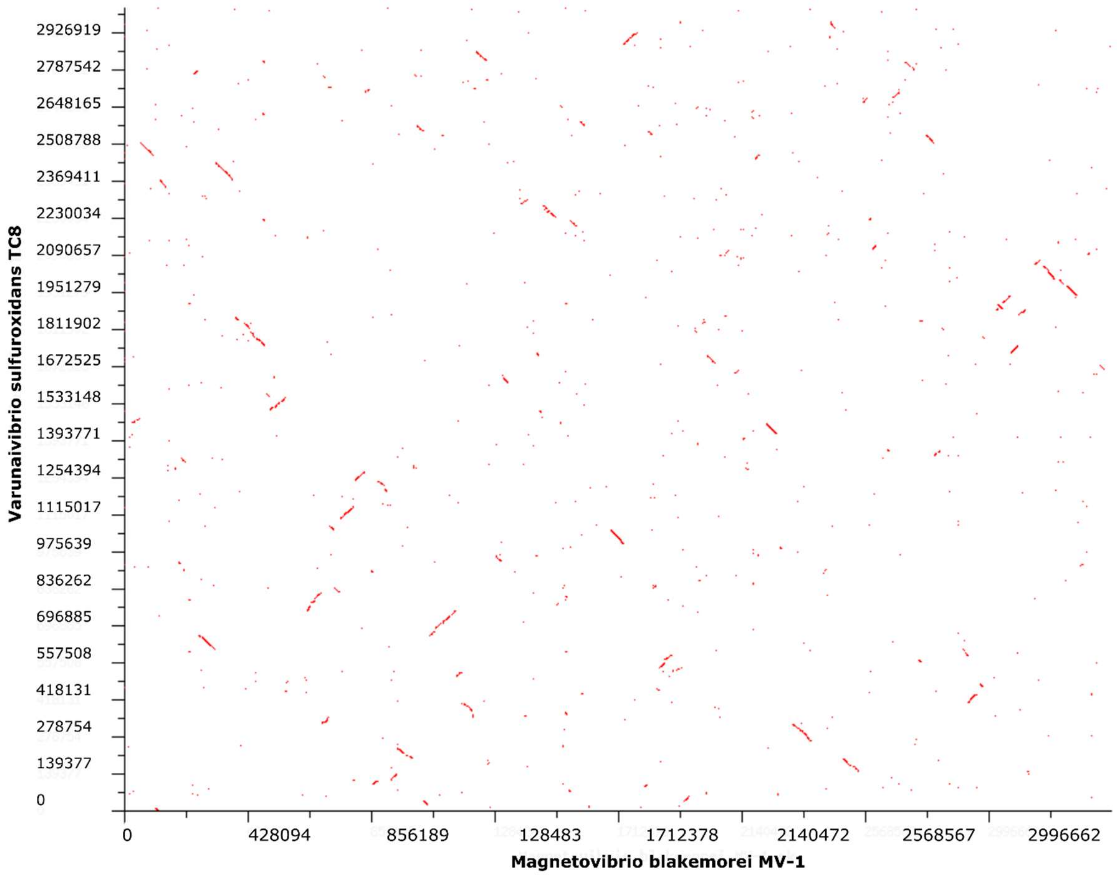

Figure S2. Blast Dot Plot analysis of *Varunaivibrio sulfuroxidans* strain TC8^{T} vs. *Magnetovibrio blakemorei* strain MV-1^{T}.
